# Established and new horizons in radiotherapy for breast cancer

**DOI:** 10.1177/17588359231161415

**Published:** 2023-03-18

**Authors:** Trudy C. Wu, Susan A. McCloskey

**Affiliations:** Department of Radiation Oncology, University of California, Los Angeles, Los Angeles, CA, USA; Department of Radiation Oncology, University of California Los Angeles, 200 Medical Plaza Driveway, Suite #B265, Los Angeles, CA 90095, USA

**Keywords:** breast cancer, breast conservation therapy, hypofractionation, post-mastectomy radiation, radiotherapy, ultrahypofractionation

## Abstract

Modern advances in diagnostics, surgery, systemic therapies, and radiotherapy (RT) have drastically revolutionized treatment strategies for breast cancer. This review outlines current and evolving treatment paradigms for RT in the breast-conserving therapy and post-mastectomy setting. In early-stage breast cancer, there is active investigation in expanding eligibility for omission of RT in women with more biologically favorable tumors and growing options to effectively irradiate less breast tissue and shorten RT treatment courses. For locally advanced breast cancer, we discuss several patient cohorts in which the necessity of post-mastectomy RT (PMRT) is commonly debated. Ongoing efforts to better refine indications for PMRT and evaluate the feasibility of hypofractionated PMRT are being studied. Metastasis-directed therapy with ablative RT is an emerging topic of interest in many cancers, including its role and impact in oligometastatic breast cancer. In this review, we will discuss the rationale for current standard of care and address in greater detail the aforementioned concepts.

## Introduction

Breast cancer remains the most common cancer diagnosis and second leading cause of death among women in 2021.^1^ Incidence continues to rise affecting more lives each year. Modern advances in diagnostics, surgery, systemic therapies, and radiotherapy (RT) have drastically revolutionized current treatment strategies compared to historical standards. In this review, we will discuss the past, present, and future directions of breast RT in the post-lumpectomy, post-mastectomy, and metastatic settings.

## Post-lumpectomy radiation therapy

RT has allowed for the evolution of locoregional curative management from disfiguring radical mastectomy and potentially morbid complete axillary lymph node dissection (ALND) to breast preservation and sentinel node biopsy (SNB), a more patient and quality of life centric approach. Several randomized trials have established the equivalence of mastectomy and breast-conserving surgery (BCS) followed by RT for management of the breast,^2,3^ and of ALND and SNB for management of the axilla in the setting of limited node positivity.^4,5^

Historically, the standard to follow BCS with adjuvant RT for early-stage invasive breast cancer was established by numerous seminal randomized trials conducted in the 1980s and 1990s. The Early Breast Cancer Trialists’ Collaborative Group (EBCTCG) published their first (of two) systematic reviews and meta-analyses in 2005 which served to quantify the impact of RT. In an analysis of over 42,000 women, adjuvant whole breast irradiation (WBI) was found to reduce the risk of local recurrence (LR) from 23% to 7% after BCS. More importantly, for every four LR averted with RT, this translated into the prevention of one breast cancer-related death. In the second updated EBCTCG meta-analysis published in 2011, of over 10,000 women after BCS, adjuvant WBI reduced the risk of any first recurrence (local, regional, or distant) from 35% to 19% at 10 years and translated into a breast cancer-specific mortality benefit at 15 years.^2^ RT was found to have the greatest absolute benefit in younger patients, in patients with large and high-grade tumors, and in those not receiving adjuvant endocrine therapy. Results of these two meta-analyses reinforced the importance of adjuvant RT and characterized those with early-stage invasive disease who were likely to benefit the most from RT.

In the trials informing the EBCTCG meta-analyses, RT was uniformly delivered daily to the whole breast over 4–6 weeks. As breast cancer screening techniques have improved, our understanding of breast cancer biology has evolved, and RT techniques become more advanced, opportunities have arisen to personalize postoperative RT management, rather than the ‘one size fits all’ paradigm of BCS and adjuvant standard fractionation WBI.

### Can RT be omitted following BCS?

The first question we currently ask when seeing women after BCS is whether RT can be omitted altogether. Modern studies have attempted to identify subsets of women in whom recurrence rates are so low that RT can be safely omitted. Women of advanced age typically develop more biologically favorable breast cancers and given decreased overall life expectancy with age, may be less likely to benefit from adjuvant RT in their lifetime. CALGB 9343 randomized a subset of patients over the age of 70 with estrogen receptor (ER)-positive tumors less than 2 cm in size to adjuvant RT plus endocrine therapy *versus* endocrine therapy alone.^6^ PRIME II did the same with women over the age of 65 with ER-positive tumors less than 3 cm in size.^7^ Findings from CALGB 9343 demonstrated a reduction in 10-year LR rate from 10% to 2% with the addition of RT^6^ (10% and 1%, respectively, in PRIME II^8^); however, no difference in overall survival (OS) was detected in either study. Given the small incremental local control benefit and lack of survival difference, endocrine therapy alone following BCS is an appropriate consideration in select women over the age of 65 with clinically node negative, ER-positive tumors less than 3 cm. While PRIME II supports consideration of omission of RT in patients aged 65–69, the NCCN guidelines follow CALGB 9343’s age criteria of age 70 or older.^9^ Several additional trials are investigating the omission of adjuvant RT in younger women with more favorable biology as evidenced by low Ki67 and/or low Oncotype DX recurrence score (Table 1). For example, the LUMINA trial prospectively omitted RT in a subset of women over the age of 55 with stage I, grade 1–2, Ki67 < 13.25%, luminal A breast cancer who had undergone BCS and were receiving endocrine therapy.^10^ Among 500 women enrolled, cumulative 5-year LR and OS were 2.3% and 97.2%, respectively. The data are awaiting publication but suggest patient eligibility for omission of RT following BCS is likely to expand.

**Table 1.** Select accruing/closed trials in early-stage invasive breast cancer.

	Premise	Inclusion criteria	Study design
LUMINA	Omission criteria after BCS may be expanded to younger women given low risk of in-breast tumor recurrence in select patients with favorable disease features	⩾55 years, pT1N0, luminal A, ER or PR+, HER2−, −LVSI, Ki67 < 13.25%	Single-arm prospective cohort study of BCS followed by endocrine therapy alone for 5 years
UK PRIMETIME	Use of the ‘IHC4 + C’ score to risk stratify women appropriate for omission	⩾60 years, pT1N0, luminal A, G1 or 2, ER or PR+, HER2−	Very low risk per IHC4 + C (endocrine only) *versus* low/int/high risk per IHC4 + C (RT + endocrine)
PRECISION	Use of the Prosigna test (PAM50) to risk stratify women appropriate for omission	50–70 years, pT1N0, ER or PR+, HER2−, G1 or 2	Low risk per PAM50 (endocrine only) *versus* int/high risk per PAM50 (RT + endocrine)
EXPERT	Use of PAM50 to define early-stage disease that may be amenable for omission	⩾50 years, pT1N0, ER or PR+, HER2−, G1 or 2, luminal A per PAM50, ROR score ⩽ 60	Randomized phase III non-inferiority trial to RT + endocrine *versus* endocrine only
NRGBR007 (DEBRA)	Use of Oncotype Dx to define early-stage disease that may be amenable for omission	50–70 years, pT1N0, ER or PR+, HER2−, Oncotype Dx ⩽ 18	Randomized phase III non-inferiority trial to RT + endocrine *versus* endocrine only
EUROPA	Adjuvant PBI may result in superior HRQoL than endocrine only after BCS in older women	⩾70 years, pT1N0, luminal A, any grade if pT ⩽ 10 mm	Randomized to PBI only *versus* endocrine only
RTOG 1005	Hypofractionated WBI with concurrent tumor bed boost is non-inferior to the standard regimen	Pathologic stage I-II, high-grade DCIS in <50 years old, yp stage I-II after BCS	Randomized phase III non-inferiority trial to standard WBI with sequential boost *versus* hypofractionated WBI with concurrent boost

BCS, breast-conserving surgery; DCIS, ductal carcinoma in situ; ER, estrogen receptor; HRQoL, health-related quality of life; PBI, partial breast irradiation; PR, progesterone receptor; RT, radiotherapy; WBI, whole breast irradiation.

Another subset of women in whom omission of RT following BCS may be considered is in patients with pure ductal carcinoma in situ (DCIS). RT has been shown to consistently decrease in-breast recurrence by 50% in the setting of pure DCIS, and is endorsed by NCCN as a category 1 guideline recommendation to follow BCS.^9^ However, recent studies have attempted to identify subsets of patients with DCIS who are sufficiently low risk to consider omission of RT. ECOG E5194 prospectively studied omission of RT for margin-negative DCIS.^11^ Among 561 patients included in the study with low–intermediate grade DCIS spanning less than 2.5 cm with negative (⩾3 mm) margins, the 12-year LR rate was 14% without RT, suggesting omission of RT may be acceptable in this select subset. However, when evaluating 105 study participants with high-grade DCIS spanning less than 1 cm with negative margins, the 12-year LR rate without RT was significantly higher at 25%. RTOG 9804 was a randomized trial evaluating BCS *versus* BCS + RT among women with low–intermediate grade DCIS spanning less than 2.5 cm with negative (⩾3 mm) margins. The 15-year results demonstrated an in-breast recurrence risk reduction from 15.1 to 7.1% with the addition of RT, although overall recurrence risk was low in both arms.^12^ RT has not been associated with a survival benefit for DCIS and is intended to limit LR risk. These data suggest that, as is the case with invasive disease, recurrence risk among women with DCIS presents on a spectrum and requires individualized decision-making with careful assessment of risks, benefits, and patient preference. It is our practice to discuss the option of omission of radiation for women over the age of 65 presenting with stage I ER+ invasive breast cancer when endocrine therapy is planned, as well as, for women with low–intermediate grade DCIS spanning < 2.5 cm with ⩾3 mm margins.

### Is accelerated partial breast irradiation an option?

As we continue to refine opportunities for omission, RT in some form remains the widely accepted standard of care for most women undergoing BCS. When a patient meets indications for RT, the first question we ask is whether she is a candidate for accelerated partial breast irradiation (APBI). This technique operates under the rationale that microscopic cells (if present) are most likely to reside near the surgical bed, and thus restricts RT exposure to a narrow margin (typically 1–2 cm) around the lumpectomy site. In addition to being advantageous in limiting dose to critical normal organs in close proximity to the breast (e.g. heart and lung), APBI also allows for abbreviated courses of RT, typically ranging between 1 and 10 fractions. To be a candidate for APBI, select pathologic (defined by the American Society for Radiation Oncology [ASTRO] consensus statement)^13^ and anatomic (well-defined and identifiable lumpectomy cavity that is of appropriate size ratio with respect to the breast) criteria ideally should be met. There are a variety of techniques to deliver APBI including intraoperative RT (IORT), intracavitary or interstitial brachytherapy, or external beam RT (EBRT). IORT is currently recommended only in the context of clinical trials given inferior outcomes published in randomized trials.^14,15^ However, there is ongoing investigation to establish whether equivalence may be achieved with IORT in highly selected subsets.

Intracavitary and interstitial brachytherapy are well-established options for the delivery of APBI; however, external beam APBI, which is less operator dependent and more widely available, has been the greater focus in recent randomized phase III trials.^16–18^ The University of Florence, RAPID, and NSABP B39/RTOG 0413 trials all evaluated APBI *versus* WBI (with EBRT), however differed in RT technique (3D conformal radiation [3DCRT] *versus* intensity-modulated radiation therapy [IMRT]) and fractionation schema. The largest of these trials, NSABP B39/RTOG 0413, included EBRT (3DCRT 38.5 Gy in 10 fractions BID) and brachytherapy (34 Gy in 10 fractions) in their APBI arm. Patients were randomized to APBI or WBI (50 Gy in 25 fractions ± sequential lumpectomy cavity boost) with in-breast tumor recurrence (IBTR) as the primary outcome. At 10 years, the authors were unable to claim non-inferiority between APBI and WBI; however, 10-year IBTR was 4.6% and 3.9% in the APBI and WBI groups, respectively. This <1% absolute difference, although statistically significant, is of questionable clinical significance.^17^ More recently, 10-year follow-up from the Florence Trial was published, which randomized women to standard fractionation WBI over 5 weeks *versus* 30 Gy in 5 every other day fractions of APBI using IMRT.^18^ Among 420 women enrolled, 10-year statistical equivalence was reported for IBTR, OS, and disease-free survival (DFS) between the two arms. Furthermore, APBI was associated with less acute and late toxicity, as well as improved cosmesis as evaluated by physician and patient. This regimen from the Florence Trial is now endorsed as the preferred APBI dose fractionation schema per NCCN.^9^ The 2017 ASTRO consensus guideline can be referenced for APBI eligibility criteria with the ‘suitable’ subset defined as age ⩾ 50, negative surgical margins ⩾ 2 mm, Tis, or T1 disease.^13^ In our practice, APBI is considered for all women meeting the ASTRO consensus criteria and the preferred regimen is the five fraction Florence Trial regimen.

### Can WBI be delivered in fewer fractions?

At its inception, external beam APBI was applauded for shortening RT treatment time and patients were able to complete adjuvant EBRT in as few as five fractions; however, the adjective ‘accelerated’ in APBI is now grossly, a misnomer. Modern efforts have attempted to improve the convenience of RT by increasing the dose per fraction while reducing the number of total fractions to maintain a biologically effective dose. This has led to shorter courses of RT to the whole breast (when compared to the historical standard of up to 7 weeks of daily RT) for many women who are not appropriate candidates for APBI. Four randomized trials with 10 years of follow-up have established the equivalence of a 3-week hypofractionated course of WBI to the standard fractionation regimen of 5 weeks.^19–21^ The comparable cosmetic outcomes between the two fractionation regimens without compromise in oncologic outcomes led ASTRO to update clinical guidelines in 2018, and strongly recommend hypofractionation as the new standard for all patients receiving WBI alone.^22^

More recently, investigators queried whether the number of fractions can be further reduced, yet maintain safety, cosmesis, and therapeutic quality. The concept of ultrahypofractionation was spearheaded in the mid-2000s by the UK FAST Trialists Group when they started enrollment for their first (of two) randomized controlled trials (RCTs).^23,24^ The premier UK FAST trial studied cosmetic outcome as the primary endpoint in patients receiving ultrahypofractionation to 28.5 Gy or 30 Gy in five once weekly fractions; and concluded a total dose of 28.5 Gy had less breast edema and shrinkage after RT (compared to 30 Gy; *p* < 0.05). At 10-year follow-up, breast induration was the only notable difference for patients receiving 28 Gy (compared to 50 Gy in 25 fractions; *p* < 0.05). The successor FAST-Forward Trial studied 26 Gy or 27 Gy in five daily consecutive fractions in an attempt to shorten total RT treatment time (from 5 weeks to 1 week) with the primary endpoint being IBTR. Inclusion criteria were broad (pT1-3N0-1, age ⩾ 18, negative margins) and 4096 patients were enrolled across 97 United Kingdom centers. Approximately one-third of patients were deemed ‘high risk’ (age < 50 and/or grade 3). At 5-year follow-up, local control, regional control, distant relapse, and OS were statistically equivalent across all three arms. Interestingly, women receiving 27 Gy in 5 fractions were found to have more breast distortion, shrinkage, induration, telangiectasias, and edema (compared to 40 Gy in 15 fractions; *p* < 0.05); however, 26 Gy in 5 fractions was more comparable to 40 Gy in 15 fractions in regard to toxicity with statistically significant, but numerically minimal differences in breast induration and edema. These results support ultrahypofractionation (26 Gy in 5 consecutive daily fractions) to be an appropriate regimen with excellent local control and acceptable cosmesis at 5-year post-RT. Although the outcome data for ultrahypofractionation are still fairly premature with only published 5-year follow-up, the COVID-19 pandemic has encouraged providers to shorten days on treatment (when possible) and led to a 300-fold increase in the adoption of ultrahypofractionation in the United Kingdom.^25,26^ The ultrahypofractionation regimens have been included in the latest iteration of the NCCN guidelines.^9^ We are currently routinely offering ultrahypofractionated whole breast radiation, with the caveat that the differential depth and breadth of data to support hypofractionated and ultrahypofractioned regimens is being discussed, while informing individualized patient decision-making.

In any woman receiving WBI, two final questions to address when finalizing a RT plan are whether a lumpectomy cavity boost is required, and whether regional lymphatics should be encompassed in the treatment volumes.

### In which patients should we consider a lumpectomy cavity boost?

After WBI is complete, additional RT to the tumor bed plus a margin, can sequentially follow. This is known as a lumpectomy cavity boost and is directed at tissue where recurrence is known to be greatest.^27^ The most widely cited boost trial was a randomized trial conducted through the EORTC and at its initial publication with 10 years of follow-up in 2007, administration of a boost was associated with a statistically significant reduction in IBTR among women of all ages; however, younger patients derived the most benefit. A 20-year update was published in 2017 that revealed equivalent 20-year OS (boost *versus* no boost), but a sustained reduction in IBTR with the addition of a lumpectomy cavity boost, at the expense of a slight increase in severe breast fibrosis. A separate analysis within this trial examined prognostic factors for local control, and found younger women (age < 50), those with DCIS present, and those with hormone receptor negative, high-grade tumors derived the greatest benefit from a boost.^28^ Guidelines published by professional societies including ASTRO, GEC ESTRO, and ESMO suggest the following risk factors be considered when recommending a boost: young age, high grade, close/positive margins, larger tumor size, extensive intraductal component, lymphovascular space invasion (LVSI), residual disease after neoadjuvant chemotherapy (NAC), and triple-negative disease. Individualized decision-making is appropriate and endorsed in boost utilization. Regarding technique, concluded and ongoing trials are assessing simultaneous integrated boost (SIB), also known as a concomitant boost, as an alternative to the more traditional sequential boost in an effort to reduce overall treatment duration. Initial data from the IMPORT HIGH trial regarding the use of SIB suggest broadly similar cosmetic outcomes between the two boost techniques.^29^ Preliminary results in abstract form of RTOG 1005, a randomized phase III trial evaluating conventional WBI with sequential boost *versus* hypofractionated WBI with SIB, suggest non-inferiority between the two arms with respect to ipsilateral breast recurrence and toxicity at a median follow up of 7.3 years.^30,31^

### In which patients should we consider regional nodal irradiation?

The final question of whether to include regional nodal irradiation (RNI) has been extensively debated for years. Individualized RT plans can be designed to encompass the breast alone following BCS or also include regional lymphatics (levels 1, 2, and 3 axilla, supraclavicular nodes, and/or internal mammary nodes). For women with four or more positive nodes, it is the widely accepted standard of care to encompass comprehensive regional nodes in the RT plan. In women with limited node positivity, two RCTs were published simultaneously in the *New England Journal of Medicine* in 2015 addressing this issue. MA.20 enrolled women undergoing BCS who had node-positive or high-risk node-negative disease (defined as >5 cm primary or >2 cm primary with either grade 3, ER−, or positive LVSI) to WBI only or WBI plus comprehensive RNI.^32^ EORTC enrolled women undergoing BCS (76%) or mastectomy (23%) who had externally located node-positive disease (56%) or centrally/medially located node-positive or node-negative disease (44%) to WBI only or WBI plus comprehensive RNI.^33^ In both trials, comprehensive RNI was associated with a small, but statistically significant improvement in 10-year DFS. Acknowledging the heterogeneity of this group and small <5% absolute benefit in DFS reported in these trials, the NCCN, ASTRO, ASCO, and SSO guideline statements suggest strong consideration of RNI in women with limited node positivity but leave room for physician discretion in ultimate decision-making. The use of an Oncotype Dx Recurrence Score to help risk stratify women with limited node positivity (1–3 nodes positive) into receiving RNI is currently under investigation in the randomized phase III trial, MA.39 TAILOR RT (A Randomized Trial of Regional Radiotherapy in Biomarker Low-Risk Node Positive and T3N0 Breast Cancer).^34^

The delivery of adjuvant RT to complete BCT has evolved into a menu of options – of variable dose, fractionation schema, and treatment volumes. The progression of conventional fractionation to hypofractionation and now, ultrahypofractionated WBI showcases the technological advances and increased confidence to safely deliver higher doses per fraction. Furthermore, efforts to avoid overtreatment of breast volumes have manifested in APBI. In the setting of WBI planning, several technological advances have also served to optimize treatment planning leading to greater dose homogeneity (i.e. evenness of dose distribution) and conformality (i.e. shaping of dose to target and sparing adjacent normal tissues). Prone breast treatment planning serves to displace the breast from the body, thus limiting exposure to the underlying lung. Deep inspiratory breath hold (DIBH) is a widely utilized technique to pull the heart back, down, and away from the breast, thus limiting its exposure to RT. Multifleaf collimators allow for precise beam shaping and minimization of lung and heart exposure. Dose homogeneity techniques include field in field and electronic compensation. Proton beam radiation therapy is being actively studied to further optimize normal organ avoidance in the setting of RNI. Many considerations such as pathologic features, toxicity risk, logistics, and most importantly, patient preference should be unified in shared decision-making and selection of a customized RT treatment plan.

## Post-mastectomy radiation therapy

In the 1980s, three seminal RCTs (the Danish 82b, Danish 82c, and British Columbia Trials), demonstrated a local control (LC), DFS, and OS benefit for post-mastectomy RT (PMRT) among women with T3-4 tumors and/or node-positive disease, thus establishing PMRT as standard of care for these select subsets.^35–37^ However, with improvements in surgical and imaging techniques, systemic therapies, and our understanding of tumor biology, the necessity of PMRT in some potentially more favorable subsets has become controversial. The primary areas of debate are among women with T3N0 disease, T1-2N1 disease, and in women receiving NAC prior to mastectomy. We will address these controversial areas below.

### Do patients with T3N0 disease need post-mastectomy radiation therapy?

The Danish and British Columbia randomized trials of PMRT demonstrated a significant decrease in the risk of locoregional recurrence (LRR) which translated into a significant survival benefit with the addition of PMRT primarily among women with large primary tumors exceeding 5 cm and/or node positive disease. In the Danish 82b and 82c RCTs, approximately 135 patients had T3N0 disease, and RT significantly reduced LRR (17% to 3% in 82b and 23% to 6% in 82c) which improved 10-year DFS in 82b (70% *versus* 82%). However, subsequent analyses have suggested much lower rates of LRR in the absence of PMRT for this subset, ranging from 7.6% to 11% across four retrospective reviews.^38–41^ What further challenges our understanding is that SEER Medicare Analyses and National Cancer Database Analyses (which do not report LRR data), note a significant OS benefit associated with PMRT for women with pT3N0 disease.^41–43^ With such heterogeneous data, the NCCN guidelines endorse ‘consider RT’ for T3N0 disease and individualized decision-making is advised. Although PMRT is not traditionally recommended for women with T1-2N0 disease, there are reports that suggest exceptions to this rule in women with additional high-risk features including grade 3, LVSI, T2 primary, triple-negative biology, and/or absence of systemic therapy. A retrospective review analyzing 10-year LRR among 1505 women with T1-2N0 disease undergoing mastectomy reported >20% LRR in the absence of PMRT for women with both grade 3 disease and LVSI and for women with grade 3, LVSI, T2 primary, and no systemic therapy.^44^ A prospective study was also conducted in China randomizing 681 patients with stage I-II triple-negative breast cancer (>80% were node negative) to chemotherapy ± PMRT.^45^ PMRT was associated with statistically significant improvement in 5-year recurrence-free survival and OS. Factors we routinely consider in advising women with pT1-3N0 disease regarding the need for PMRT include age/menopausal status, comorbidities/life expectancy, tumor size, margin status, LVSI, molecular subtype, tumor grade/Ki67, anatomic tumor location, receipt of systemic therapy, toxicity risks (e.g. extent of lymph node [LN] surgery, reconstruction, and laterality with respect to cardiac risk), and patient preference.

### Do patients with limited node positivity (N1, 1–3 positive nodes) need post-mastectomy radiation therapy?

The aforementioned Danish and British Columbia RCTs reported a LRR and OS benefit associated with PMRT among women with any number of positive nodes, and found that in women with N1 disease (1–3 nodes positive) specifically, 10-year cumulative rates of LRR were approximately 30% in the absence of PMRT.^35–37^ In subsequent studies that included women undergoing more extensive axillary surgeries and/or receiving more modern systemic therapies, 10-year cumulative rates of LRR were lower, in the teens. To clarify this discrepancy, a subset analysis of the Danish 82b and 82c trials was performed and limited to only women with N1 disease who had at least eight nodes removed surgically. Among 1152 women analyzed with N1 disease, at 15 years of follow-up, LRR was 27% without RT and 4% with RT. This translated into a 9% statistically significant absolute OS benefit at 15 years.^46^ The Early Breast Cancer Trialists Group conducted a meta-analysis including 8135 node positive patients across 22 trials and similarly reported a statistically significant reduction in 10-year LRR (20.3% without RT and 3.8% with RT), that translated into an absolute 8% decrease in 20-year mortality among women receiving PMRT.^47^

Despite this compelling data from RCTs and subsequent subset and meta-analyses, there remains hesitancy in the applicability of these findings in the modern era where significant improvements in imaging, surgery, and systemic therapy have been made. For example, investigators at MD Anderson retrospectively compared LRR among women with T1-2N1 disease receiving and not receiving PMRT between the years of 1978 and 1997 *versus* 2000 and 2007, and found no benefit to those receiving PMRT in the modern era.^48^ Several other retrospective series have attempted to identify specific conglomerate risk factors that impact LRR in an effort to better specify PMRT indications. A retrospective review conducted at MSKCC identified the combination of age < 50 and LVSI as predictive for higher LRR risk in the absence of PMRT.^49^ Tumor biology as assessed by the 21 gene recurrence score has also been shown to be predictive for LRR.^50–52^ In 2016, ASTRO, ASCO, and SSO released a guideline statement regarding PMRT, and unanimously agreed that PMRT reduces LRR and breast cancer mortality among women with T1-2N1 disease; however, the absolute benefit of PMRT varies within this heterogenous cohort and the panel endorsed a balanced approach using clinical judgment and consideration of risks and benefits to reach an individualized decision for each patient.^53^ The NCCN guidelines endorse ‘strongly consider RT’ for T1-2N1 disease.^9^ Factors we routinely consider include those mentioned above for T3N0 disease (age/menopausal status, comorbidities/life expectancy, tumor size, margin status, LVSI, molecular subtype, tumor grade/Ki67, anatomic tumor location, receipt of systemic therapy, toxicity risks [e.g. extent of LN surgery, reconstruction, and laterality with respect to cardiac risk], and patient preference), in addition to extent of LN surgery, number of nodes positive, nodal burden within the node(s), and extra nodal extension. This matter is under active investigation in the MA.39 TAILOR RT Trial and SUPREMO RCTs (Table 2).^34,54^

**Table 2.** Select accruing/closed trials in locally advanced breast cancer.

	Premise	Inclusion criteria	Study design
SUPREMO	The addition of PMRT does not have a meaningful impact on survival in an intermediate risk group of women after mastectomy (±chemo)	pT1-2N1, pT3N0, pT2N0 with G3, and/or LVSI	Randomized phase III trial to PMRT *versus* no PMRT
TAILOR RT/MA.39	There may be a subset of women with limited node positivity or cT3N0 that may not benefit from adjuvant RT	T3N0, T1-2N1 with 1–3 positive LN after ALND or 1–2 positive LN after SLNB, ER or PR+, HER2−, Oncotype Dx ⩽ 25, ⩾35 years	Randomized phase III trial to adjuvant RT *versus* no adjuvant RT
RT CHARM	Hypofractionated PMRT is non-inferior to fractionated PMRT for mastectomy with reconstruction	Inclusion of RNI, stage IIA–IIIA, planned reconstruction	Randomized phase III non-inferiority trial to fractionated PMRT 50 Gy in 25 fx *versus* hypofractionated PMRT 43.5 Gy in 15 fx
NSABP B51/RTOG 1304	Whether the addition of PMRT + RNI after mastectomy or the addition of RNI after BCS improves outcomes in ypN0 women	cT1-3N1, ypN0(i+) or ypN0(mol+), 8 weeks of anthracycline, and/or taxane-based chemo (±HER2-directed therapy)	Randomized phase III to PMRT + RNI *versus* no PMRT after mastectomy or RNI *versus* no RNI after BCS

ALND, axillary lymph node dissection; BCS, breast-conserving surgery; ER, estrogen receptor; LN, lymph node; PMRT, post-mastectomy RT; PR, progesterone receptor; RNI, regional nodal irradiation; RT, radiotherapy.

### Do patents need post-mastectomy radiation therapy after NAC?

Another topic of controversy surrounds PMRT in women undergoing mastectomy following receipt of NAC. Whether we can tailor RT recommendations to chemotherapy response is an area of active debate and study. Women receiving NAC are a remarkably heterogeneous group and the data currently available to guide decision-making are entirely retrospective. There is a general consensus that PMRT is indicated for women with cT3-4, cN2-3, and/or residual node-positive disease after the receipt of NAC. This is based on retrospective data from the MDACC and NSABP clinical trials. A MDACC retrospective analysis of LRR risk among women receiving NAC and mastectomy with and without PMRT suggested significant differences among patients with cN2-3 disease (10-year LRR 40% without PMRT and 12% with), clinical stage III disease and pathologic complete response (pCR) (10-year LRR 33% without PMRT and 7.3% with), age < 35 with clinical stage II or III disease (5-year LRR 37% without PMRT and 12% with), and cT3N0 disease (5-year LRR 24% without PMRT and 4% with).^55^ The NSABP conducted a retrospective analysis of 10-year LRR among women enrolled in the NASBP B-18 and B-27 prospective trials of NAC without PMRT.^56^ When categorized by initial tumor size, clinical nodal status, and presence or absence of residual disease in the breast or nodes after NAC, women who had residual nodal disease had a 10-year LRR exceeding 10% regardless of all other factors. Based on this data, PMRT is the widely accepted standard of care for women with cT3-4, cN2-3, and/or residual node-positive disease after the receipt of NAC.

The major area of controversy is among women with clinical T1-2N1 disease who convert to node negative after NAC. The aforementioned MDACC retrospective analyses and NSABP trial data analyses assessed outcomes among women with cT1-2N1 disease who achieved a pCR with NAC. In both series, 10-year LRR was 0%; however, there were very few patients included in the analysis (*n* = 20 and *n* = 21 in the MDACC and NSABP series, respectively). The German Breast Cancer Trialists Group analyzed data from three prospective neoadjuvant trials in which 82.7% of participants received PMRT. On multivariate analysis, women with node positive disease benefitted from PMRT regardless of response. Among the node-positive subset converting to node-negative disease, 5-year LRR with and without PMRT was 9.3% *versus* 22.2%, respectively (*p* = 0.05).^57^ A retrospective NCDB analysis conducted among clinically node-positive women who converted to clinically node negative after NAC found PMRT to be beneficial among those with clinical stage IIIB-C disease, clinical T3-4 disease, and those with residual disease in the breast.^58^ The NCCN guidelines currently endorse ‘strongly consider RT’ for those with clinically node-positive disease who convert to node negative.^9^ We eagerly await results from NSABP B-51 which randomized women undergoing mastectomy who convert from node-positive to node-negative disease after NAC to PMRT or omission of PMRT (Table 2).^59^

### Can post-mastectomy radiation therapy be delivered in fewer fractions?

Although hypofractionation is the new norm to follow BCS, standard fractionation over approximately 5 weeks remains the standard of care in the post-mastectomy setting. The reluctance to adopt hypofractionation among women receiving PMRT is primarily due to concerns over increased toxicity risk from dose exposure to the heart, lung, and/or brachial plexus, as well as cosmesis concerns in the setting of breast reconstruction. This topic is currently under extensive investigation with at least five randomized trials evaluating the efficacy and toxicity of hypofractionated PMRT, including the aforementioned MA.39 TAILOR RT Trial.^34,60,61^

## Metastatic breast cancer

In the setting of non-metastatic breast cancer, locoregional therapies including surgery and RT are used to treat macroscopic localized disease in the breast and regional nodes while systemic therapies are utilized primarily to treat microscopic disease beyond the breast and nodes. In the metastatic setting, we have traditionally relied exclusively on systemic therapy, reserving RT for symptom palliation. In recent years, there has been growing interest in the role of locoregional therapies, such as RT, to treat macroscopic metastatic disease with ablative intent. In 1995, Hellman characterized the concept of an oligometastatic state as a distinct entity between localized and widely metastatic disease that may be amenable to a curative therapeutic strategy.^62^ The most widely accepted definition of oligometastatic disease is ⩽5 metastases, and can be categorized as *de novo* (i.e. present at initial diagnosis), oligorecurrent (i.e. develop after the delivery of definitive therapy for localized disease), induced (i.e. remain after systemic therapy), and/or oligoprogressive (i.e. develop after complete initial response to systemic therapy). Regardless of the specific circumstance, many have hypothesized that treatment of oligometastases as a supplement to standard of care systemic therapy could be beneficial.

Early data assessing the possible role of RT for oligometastatic breast cancer were promising. The University of Rochester published a series of analyses evaluating SBRT for oligometastatic disease across many cancer subtypes.^63^ Breast cancer patients rapidly emerged as having the greatest potential benefit with a final analysis reporting 10-year OS of 75% among a small subset of women with bone only metastatic breast cancer receiving SBRT to oligometastases.^64^ An Italian study of SBRT for oligorecurrent breast cancer as well as an Australian single institution series of SBRT for bone only oligometastatic disease reported 2-year PFS of 53% and 67%, respectively, which compared very favorably to the approximate 30% expected PFS in this metastatic subset without metastasis-directed therapy (MDT).^65,66^

The most promising data for SBRT in oligometastatic disease came from SABR-COMET, a phase II prospective randomized trial that evaluated the benefit of SBRT for patients with *de novo* oligometastatic cancer (<5 metastases) and a controlled primary lesion after definitive treatment.^67^ Approximately 20% of patients enrolled had a breast cancer primary. The primary endpoint was OS and with a median follow up of 51 months, SBRT was associated with a statistically significant OS, PFS, and LC benefit. There was an impressive 22-month median OS benefit associated with MDT of 28 months *versus* 50 months in the standard of care and SBRT arms, respectively. SABR-COMET set the stage for future studies of SBRT for oligometastases in breast cancer specifically. The first randomized prospective trial specific to breast cancer patients with oligometastases was NRG BR002, a prospective phase II trial that enrolled women with locally controlled metastatic breast cancer with ⩽4 metastatic sites to standard of care systemic therapy *versus* standard of care systemic therapy plus ablative RT to all oligometastases.^68^ Results were presented at ASCO 2022. Among 125 women enrolled, median age was 54, 79% were ER+ or progesterone receptor positive and HER2− (8% triple negative), 60% had one oligometastasis, and 93% received ablative SBRT to all oligometastases. At a median follow-up of 30 months, PFS and OS were equivalent with no additional benefit after ablative RT to metastatic sites. Given this finding, a planned phase III NRG trial will not proceed. Similarly, another negative phase II trial (Consolidative Use of Radiotherapy to Block Oligoprogression [CURB]) evaluated the role of SBRT in patients with oligoprogressive metastatic non-small-cell lung cancer (NSCLC) or breast cancer, and only detected a PFS benefit in the NSCLC cohort, and not in women with breast cancer.^69^ Although these data fail to show benefit associated with SBRT among breast cancer patients with oligometastatic disease, this population is remarkably heterogeneous and future efforts to identify specific subsets who may benefit from SBRT to oligometastases are likely to continue (Table 3). In our practice, acknowledging the results of NRG BR002 and CURB which failed to show benefit for SBRT to oligometastases, we take a highly selective approach in the utilization of RT factoring in histology, overall disease burden, location of oligometastases and associated symptoms, risk of progression, systemic response to date, and systemic options.

**Table 3.** Select accruing/closed trials in metastatic breast cancer.

	Premise	Inclusion criteria	Study design
OLIGOMA	The addition of MDT with RT to systemic therapy in oligometastatic breast cancer improves PFS and QOL	Metastatic breast cancer with ⩽5 metastases (includes intra-cranial)	Randomized to systemic therapy *versus* systemic therapy + MDT with RT
JCOG1017/PRIM-BC	Systemic therapy + primary tumor resection in *de novo* metastatic breast cancer is superior to systemic therapy alone	*De novo* metastatic breast cancer treated with 3 months of primary systemic therapy	Randomized to continuation of systemic therapy *versus* primary tumor resection + systemic therapy

MDT, metastasis-directed therapy; PFS, progression-free survival; QOL, quality of life; RT, radiotherapy.

## Conclusion

Breast cancer continues to impact the lives of many women globally. Advances in detection, surgery, radiation, and systemic therapy have drastically improved therapeutic options for patients, resulting in better prognoses and quality of life. General movement in the field of radiation oncology to increase dose per fraction safely and effectively, shorten RT treatment time, and minimize toxicity have been applied in breast cancer during the current era. This has resulted in modernized treatment paradigms which have widely been accepted as standard of care and paves the way to redefine novel paradigms in the future.

## Declarations

**Ethics approval and consent to participate:** Not applicable.

**Consent for publication:** Not applicable.

**Availability of data and materials:** Not applicable.
